# Preparation of PLGA Microspheres Using the Non-Toxic Glycofurol as Polymer Solvent by a Modified Phase Inversion Methodology

**DOI:** 10.3390/polym16030434

**Published:** 2024-02-04

**Authors:** Douglas Sobel, Barath Ramasubramanian, Puja Sawhney, Keerat Parmar

**Affiliations:** Medical School, Georgetown University, Washington, DC 20057, USA; br232@georgetown.edu (B.R.); ps1097@georgetown.edu (P.S.); kp705@georgetown.edu (K.P.)

**Keywords:** PLGA, glycofurol, phase inversion, microsphere, dexamethasone, drug release, gels

## Abstract

Poly(D,L-lactide-co-glycolide is a biodegradable copolymer that can release pharmaceuticals. These pharmaceuticals can provide local therapy and also avert the clinical issues that occur when a drug must be given continuously and/or automatically. However, the drawbacks of using poly(D,L-lactide-co-glycolide include the kinetics and duration of time of poly(D,L-lactide-co-glycolide drug release, the denaturing of the drug loaded drug, and the potential clinical side effects. These drawbacks are mainly caused by the volatile organic solvents needed to prepare poly(D,L-lactide-co-glycolide spheres. Using the non-toxic solvent glycofurol solvent instead of volatile organic solvents to construct poly(D,L-lactide-co-glycolide microspheres may deter the issues of using volatile organic solvents. Up to now, preparation of such glycofurol spheres has previously met with limited success. We constructed dexamethasone laden poly(D,L-lactide-co-glycolide microspheres utilizing glycofurol as the solvent within a modified phase inversion methodology. These prepared microspheres have a higher drug load and a lower rate of water diffusion. This prolongs drug release compared to dichloromethane constructed spheres. The glycofurol-generated spheres are also not toxic to target cells as is the case for dichloromethane-constructed spheres. Further, glycofurol-constructed spheres do not denature the dexamethasone molecule and have kinetics of drug release that are more clinically advantageous, including a lower drug burst and a prolonged drug release.

## 1. Introduction

New technologies to deliver drugs continuously and automatically [1,2,3,4] have been developed to circumvent drug delivery problems in the treatment of systemic and local disease. The problems of poor medication compliance and wide hazardous swings in drug levels particularly occur when repeat parenteral or enteral administration is required.

Some of the new drug delivery technologies have included pump administration of drugs, utilization of gels, and the parenteral administration of drug laden microcrystals, lipospheres, and polymers [1,2,3,4,5,6].

PLGA, poly(D,L-lactide-co-glycolide), a composite biodegradable copolymer made up of lactic acid and glycolic acid, is of great interest as a drug releasing system [7,8]. When placed in the body, the polymer can release various kinds of substances and medications. Further, such drug release near a diseased tissue can provide local drug treatment that obviates the need for higher dose systemic therapy. This has been studied in many diseases including scleritis, glaucoma, and cancer [9,10,11]. The FDA has approved many PLGA products for human use, which include medications, growth factors, and hormones [12,13,14,15].

However, there are drawbacks of using PLGA. First, there can be issues with the kinetics of drug release. The kinetics is mostly triphasic. There is a large release (burst) of drug soon after the time of PLGA placement followed by a lag phase with little drug release prior to a more consistent drug release [16,17]. Thus, the patient may initially obtain excessive then inadequate drug treatment. Efforts have been made to solve these problems of drug release and include the embedding of drug-laden PLGA in gels and pre-incubating PLGA for days or weeks prior to use [18,19]. The second drawback is that drugs within PLGA spheres may become denatured, particularly proteins, due to the acidic environment that is generated as PLGA is hydrolyzed [20]. The third drawback is that PLGA spheres are frequently constructed with organic solvents, most commonly chloroform and dichloromethane (DCM) [16]. These solvents are classified as Class 2 drugs by the FDA, substances that should be limited because of their inherent toxicity [21].

Although approved for clinical use, side effects of currently used PLGA-laden drugs include sterile abscess, pain, and swelling. These side effects appear to be due to the organic solvents needed to dissolve the PLGA when constructing PLGA spheres [22,23]. These problems may occur even after extracting the solvent from the spheres. To avert these problems, less toxic solvents have been utilized with limited success [24,25].

One of the least toxic solvents that has been explored is glycofurol, a semi polar solvent agent used for gel formation [26,27]. Glycofurol is a good candidate as a PLGA solvent because it is nonhazardous according to the 2012 OSHA Hazard Communication Standard (29 CFR 1910.1200) [28], biocompatible, and approved for human use [29]. However, preparing drug-laden PLGA spheres with glycofurol as the solvent has had its deficiencies. One issue is that the duration of drug release has been short [30,31,32,33].

This study describes a methodology to construct dexamethasone (DEX)-laden PLGA microspheres utilizing glycofurol as the only solvent, and demonstrates that these microspheres are able to release DEX over an extended period of time. We further assess the toxicity of the spheres on target cells, the potential drug denaturing effect of these spheres, and the kinetics of drug release.

## 2. Methods and Materials

Glycofurol (Tetraglycol BioXtra Product #T3396) and PLGA with lactide:glycolide ratio of 75:25 and mol. Wt. of 66,000–107,000 (Product # P1941) was obtained from Sigma Aldrich (St. Louis, MO, USA). Dexamethasone (DEX) 98% was obtained from Alfa Aesar (Havervill, MA, USA), Product #A17590. Dichloromethane (DCM) and polyvinyl alcohol (PVA, 87–90% hydrolyzed, M_w_ 30,000–70,000) were purchased from Sigma Aldrich.

### 2.1. PLGA Microsphere Preparation

**Figure 1:**
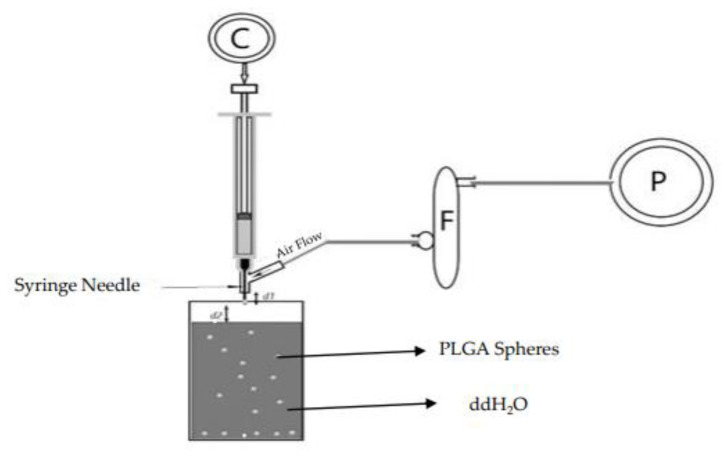
PLGA droplet generator apparatus.

PLGA spheres were prepared with glycofurol by phase inversion method (GPI) using an air-driven droplet generator device. The droplet generator apparatus was composed of syringe, syringe pump C, flow regulator F, and air pump P where d1 is the distance between the needle tip and the air flow tube and d2 is the distance between the needle tip and the surface of the water (Figure 1).

PLGA (lactide:glycolide ratio of 75:25) was suspended in undiluted glycofurol at concentrations of either 1, 5, or 20% *w/v* for Dex and 20% PLGA concentration for Sudan 111. The mixture was then placed on a rotary shaker at 150 rpm for 24 h. at room temperature to ensure complete dissolution. DEX was added to the polymer solution at concentrations of 1% and 20% (wt./wt. PLGA) and Sudan ll was added at 20% (wt/wt PLGA) The PLGA–glycofurol solution was then pumped via a Hamilton 100 μL syringe using a Hamilton syringe pump at room temperature, through a needle (27 and 30 gauge) surrounded by an air jacket into distilled deionized water. The air flow rate of 15, 30, 45 LPM with a needle exposure of 2 or 5 mm was used (Figure 1). Phase inversion quickly resulted in the formation of PLGA microspheres.

PLGA spheres remained in water at room temperature for 30 min to ensure complete phase inversion. After collected by filtration (5 μm pore cellulose membrane), spheres were washed twice with distilled deionized water, lyophilized, and then stored at −20 °C prior to use.

PLGA spheres were prepared with glycofurol using the glycofurol extraction method (GEM) as described by Allhenn [32], with the only modifications being the stirring speed to generate spheres of comparable size as those obtained using the phase inversion method described above. Briefly, 100 mg PLGA and 20 mg drug were dissolved in undiluted glycofurol at 20% *w*/*v*. This internal phase was emulsified by stirring at 200 rev./min over 8 h at 40 °C in 100 mL of 0.1% PVA, the external phase. Distilled water (200 mL) was then added slowly under stirring at 200 rev./min. The precipitated microspheres were then filtered (5 μm pore cellulose membrane filter), washed with distilled water, dried overnight under vacuum, and stored at −20 °C.

PLGA spheres were prepared with DCM using a solvent extraction technique. PLGA polymer was dissolved in DCM at 20% *w/v* and soon after DEX was dispersed in this solution at 20% *w*/*w* PLGA. This organic phase was then slowly added to 10 mL of PVA (average MW 30–70 kDa) solution (1% (*w*/*v*) under constant mechanical stirring at 250 rpm. The emulsion was then transferred to 125 mL of aqueous PVA solution (0.1% (*w*/*v*), MW 30–70 kDa) and stirred at 250 rpm under vacuum for 2.5 h to evaporate the solvent and harden the microspheres. The microspheres were then washed three times with 10 mL deionized water, collected by centrifugation, lyophilized, and stored at −20 °C.

### 2.2. Assessing Effect of PLGA Concentration on Solidification Time

The solidification time (t_1_), the time required for phase inversion to complete, was assessed and compared in 20% PLGA, 10% PLGA, 5% PLGA, and 1% PLGA spheres (n = 12/group) not containing drugs by determining the required time for spheres to turn opaque once the air-generated droplet entered the aqueous phase. To confirm that phase inversion was complete at the solidification time, some PLGA spheres were taken out of the aqueous phase immediately after turning opaque and some remained in the aqueous phase for another 5, 10, and 15 min. Following routine processing, sphere hardness was determined and compared in all groups.

### 2.3. Assessment of Sphere Hardness by Measurement of Compressive Modulus

Method to assess hardness was similar to that described by Rodriguez [34] where PLGA spheres are placed on a stage and an increasing force is exerted on top of the spheres to deform the sphere.

The original diameter (d) and cross-sectional area (A) of the sphere were noted. The force applied at which the sphere deforms by 50% was determined by the change in diameter of the sphere. From this the compressive modulus € is determined by Equation (1):

(1) $$\text{€}=\frac{\frac{Force}{Area}}{\frac{Change\;in\;diameter}{Original\;diameter}}$$

### 2.4. In Vitro Release of DEX from Microspheres

In vitro release studies were performed in phosphate-buffered saline (PBS). PLGA microspheres were placed in tubes containing 500 uL of PBS (n = 4, 15 spheres per tube) and incubated at 37 °C. At set time intervals, PBS solution was removed from the tubes for drug determination and fresh PBS was carefully replaced. Using a SPECTRAmax 340PC 384 Microplate Reader, standards were run along with the samples at 242 nm to determine the amount of drug being released from the spheres [35]. Three batches of microspheres were investigated and the means and standard deviations reported.

### 2.5. Cytotoxicity of PLGA Microspheres on Fibroblasts and Splenocytes

C57Bl/6 mouse spleen cells were isolated using Ficoll–Paque density gradient. Cells (1 × 10^5^) were mixed in a 96-well plate with Con A (2 mcg/mL). C57Bl6 mouse embryonic fibroblasts (1 × 10^5^) were pipetted in a 96-well plate and also served as target cells. Wells with spleen and fibroblast target cells were individually incubated with PLGA microspheres at 0.5, 1, 2.5, and 5 mg/mL in complete RPMI and DMEM, respectively. After 2 days of incubation, cell proliferation was assessed by Alamar Blue immunofluorescent assay, which measures mitochondrial oxidation–reduction activity using resazurin as an oxidation–reduction indicator [36].

### 2.6. Measurement of Drug Load and Encapsulation Efficiency

To determine the mass of DEX within spheres, PLGA microspheres (10 mg, 3 batches) were dissolved in 0.9 mL DMSO within a glass tube for 1 h. Then, 3 mL of 0.05 M HCl was added and incubated for another 1 h. Drug content was determined using spectrophotometric analysis [37]. The actual drug load and encapsulation efficiency was calculated by Equations (2) and (3). The theoretical drug load is the max drug load of the PLGA.

(2) $$Actual\;Drug\;Load\%\left(DL\right)=\frac{Total\;mass\;of\;drug\;in\;spheres\;(m)}{Total\;mass\;of\;spheres}\times 100\%$$

(3) $$Encapsulation\;efficiency\%\;(EE)=\frac{Actual\;drug\;load}{Theoretical\;drug\;load}\times 100\%$$

### 2.7. Determination of PLGA Microsphere Size, Density, and Porosity

The average diameters of microspheres were analyzed by software image measurement (NIH, ImageJ) [38]. The density and porosity values of the PLGA spheres were measured in triplicate by a liquid displacement methodology [39]. Forty PLGA spheres were immersed in a volumetric device containing a known volume (V1) of water. The sample was allowed to stand for 10 min and the new volume was then recorded as V2. The volume difference (V2−V1) represented the total volume of the PLGA spheres.

The density of the PLGA (ρ_p_) was expressed as ρ_p_ = W/(V2 − V1). ρ_solid_ indicates the density of the solid native PLGA. The porosity of the PLGA (φ) expressed as percentage (%) was calculated by Equation (4):

(4) $$\;\varphi (\%)=\frac{\rho_{solid}-\rho_{p}}{\rho_{solid}}\times 100\%$$

### 2.8. Assessment of Water Diffusion into PLGA Microspheres [40]

Lyophilized PLGA microspheres were weighed to obtain the dry weight (w1). The spheres were then placed in tubes containing 500 uL of ddH_2_O (n = 4, 10 mg per tube) and incubated at 37 °C. At set time intervals the spheres were removed from the water, blotted to remove excess water, and weighed to obtain the wet weight (w2). The spheres were then lyophilized to obtain the dry weight of the spheres at each time interval (w1). The % water diffusion was then calculated using Equation (5).

(5) $$\%\;Water\;Diffusion=\frac{w2-w1}{w1}\times 100$$

### 2.9. Measurement of Residual Glycofurol

**Figure 2:**
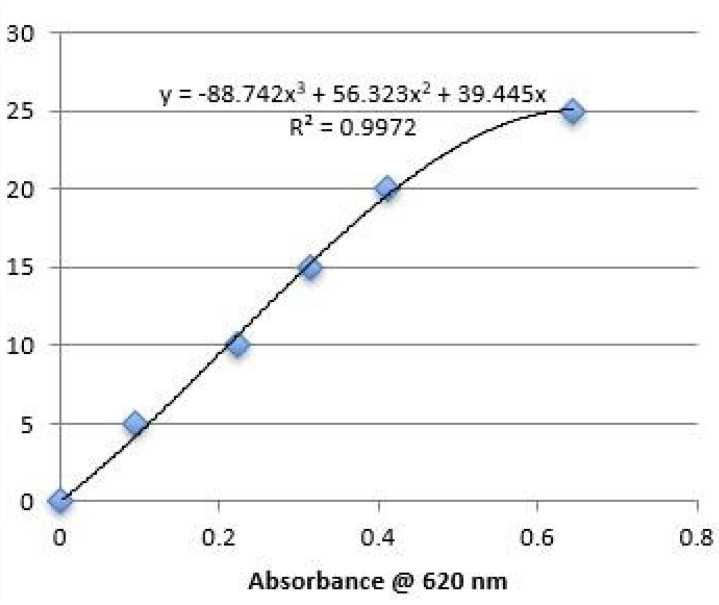
Calibration curve for glycofurol assay.

Residual glycofurol content was analyzed as described by Allhenn [32]. Blank spheres (20 mg) were dissolved in 1 mL acetone, re-precipitated in 4 mL of distilled water, and centrifuged at 6000 rpm for 30 min. A total of 4 ml of ammonium cobalt thiocyanate solution and 4 mL methylene chloride were added to 2 mL of the supernatant. The mixture was centrifuged at 3000 rpm for 10 min and the methylene chloride phase was obtained and analyzed spectrophotometrically for residual glycofurol content on a SPECTRAmax Microplate Reader at 620 nm. All experiments were performed in triplicate. The calibration curve is depicted in Figure 2.

### 2.10. Quantification of DEX Breakdown by HPLC

DEX-loaded PLGA microspheres were added to tubes containing 500 uL of PBS (n = 4, 15 spheres per tube) and incubated at 37 °C. At weekly intervals, PBS was withdrawn and replaced with fresh PBS. Specimens were assessed at 0, 1, 3, and 5 weeks by HPLC analysis for DEX and DEX breakdown products. HPLC system (Waters 600 pump; Waters, Milford, MA, USA), equipped with a UV detector (RAININ, Dynamax, Absorbance Detector Model UV-C) and a Zobax C18 (4.6 mm × 15 cm, Agilent, Santa Clara, CA, USA) analytical column were used. All samples were analyzed by isocratic method with a mobile phase containing acetonitrile/water/phosphoric acid (35/70/0.5, *v*/*v*/*v*) at a flow rate of 1 mL/min with an injection volume of 20 μL. The detector was set at a wavelength of 240 nm.

### 2.11. Assessment of the Kinetic Models of Drug Release and Statistical Analyses

Mathematical models of drug release that were tested included: first-order, Gompertz, Higuchi, Hixson–Crowell, Korsmeyer–Peppas, Weibull, and zero-order. In order to determine the kinetic model that best describes the dissolution profile of the drug-loaded PLGA system, the goodness of fit was established in each replicate using the model selection criteria (MSC), Akaike’s information criteria (AIC), mean squared error, and adjusted R^2^. The model parameters were calculated using the DDSolver program [41]. The release/diffusion mechanism was assessed by calculating the diffusion exponent A and erosion exponent B derived from the non-linear fitted Kopchas model [42].

Student *t*-test was used to determine significant differences between the means including parameters of the kinetics models under consideration. A *p* < 0.05 was considered significant.

## 3. Results

The morphology and sizes (diameter) of spheres resulting from alterations of the following device parameters: needle gauge, distance of needle exposed past below the air tube, distance of needle to aqueous surface, and air flow rates are found in Table 1. Increasing the distance of needle exposure (d1) and decreasing the air flow resulted in larger sized spheres. Certain parameters, particularly lower PLGA concentrations and higher air flow rates, yielded spindle-like PLGA rods that were not uniform and not utilized in further experiments.

The effect of altering PLGA concentrations from 1% to 20% on the solidification time and hardness (compression modulus) of the microspheres constructed with glycofurol was assessed (Table 2). Mean solidification time and mean compressive modulus were significantly different between all PLGA groups (*p* < 0.001 and *p* < 0.003, respectively). PLGA concentration correlated with compression modulus (R = 0.9919, R^2^ = 0.9839, *p* < 0.01) and inversely correlated the solidification times (R = −0.9864, R^2^ = 0.973, *p* < 0.01). To confirm that phase inversion was complete at the measured solidification times, a portion of the PLGA spheres were taken out of the aqueous phase immediately after turning opaque and some remained in the aqueous phase for another 5, 10, and 15 min prior to routine processing. Mean sphere hardness was compared within PLGA groups over time and were found not significantly different from each other.

### 3.1. Physical Properties

**Figure 3:**
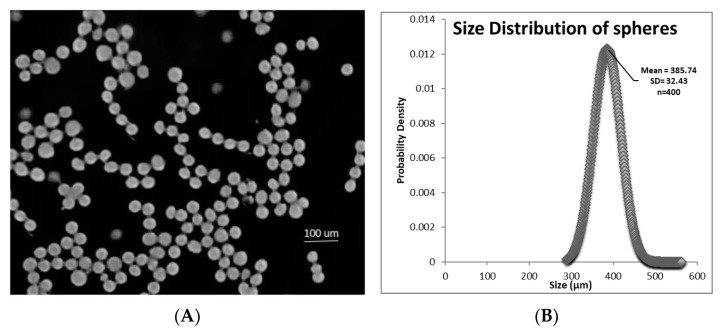
(**A**) Light photomicrograph of glycofurol-constructed spheres prepared with 20% PLGA and 20% DEX; (**B**) size distribution of spheres determined by software image measurements (ImageJ).

Utilizing 20% PLGA in glycofurol and the following device parameters: flow rate of polymer/drug through the needle of 5 µL min^−1^, air flow rate of 30 LPM, d1 at 2.5 mm, and d2 at 25 mm, the formed microspheres were spherical (Figure 3A) and had a mean diameter of 385.74 µm (±32.43 SD) (Figure 3B).

The mean glycofurol content of microspheres made from 20% PLGA was 6.26 mg (±1.78 SE) per 100 mg of PLGA microspheres.

The density, porosity, and drug burst from glycofurol phase inversion (GPI)- and DCM-constructed spheres were assessed and compared (Table 3). Sphere density and porosity constructed with GPI and DCM extraction methodologies with and without DEX were similar.

The DEX drug encapsulation efficiency within the GPI spheres were significantly greater than that found in DCM-constructed spheres while the burst was significantly lower in the GPI spheres (Table 3). GPI spheres prepared with 1% DEX had a significantly higher encapsulation efficiency than 20% DEX spheres.

The hardness of DCM- and GPI-constructed PLGA spheres (n = 30) made with 20% PLGA were compared by measuring the compressive modulus. The mean compressive modulus € of DCM spheres 21.16 (1.00) × 10^−3^ mPa was greater than GPI-constructed spheres 18.44 (0.73) × 10^−3^ mPa) *p* < 0.003.

### 3.2. Water Diffusion

**Figure 4:**
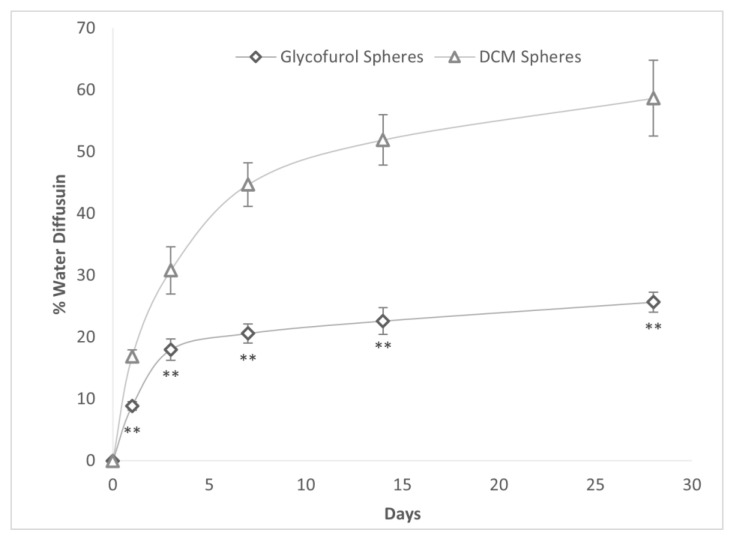
% Water diffusion into PLGA spheres constructed with either GPI method and DCM as a solvent with a PLGA concentration of 20% and drug concentration of 20%. ** *p* < 0.005 vs. DCM spheres.

The in vitro diffusion of water into DCM- and GPI-prepared PLGA microspheres laden with 20% DEX over 28 days was compared (Figure 4). The % water diffusion was significantly greater into DCM spheres than into GPI-made spheres at all time points and was more than twice as great from day 7 to the end of the study at day 28.

### 3.3. HPLC of Spheres

**Figure 5:**
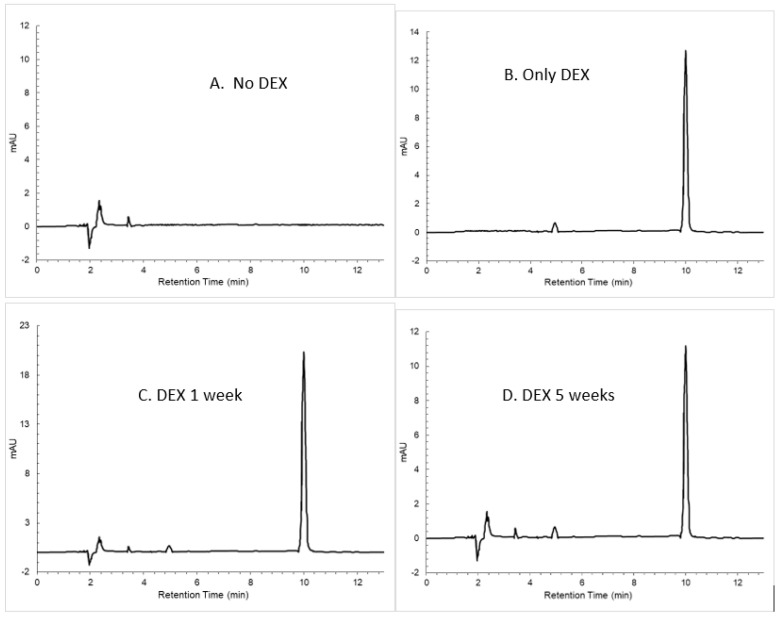
HPLC chromatogram of (**A**) PLGA without DEX, (**B**) only DEX at a concentration of 2 µg mL^−1^, (**C**) DEX-loaded PLGA spheres at 1 week, (**D**) DEX-loaded PLGA spheres at 5 weeks.

High-performance liquid chromatography was performed to determine if the DEX molecule released from the spheres is denatured over time (Figure 5). Supernatant from incubated microspheres prepared with 20% DEX and 20% PLGA within glycofurol was analyzed by HPLC. DEX had a retention time of 9.99 min. There was a sharp DEX peak at 1 week that did not alter over time. There was no secondary breakdown peak(s), even by 5 weeks.

### 3.4. In Vitro Cytotoxicity of DCM- and Glycofurol-Prepared PLGA Microspheres

**Figure 6:**
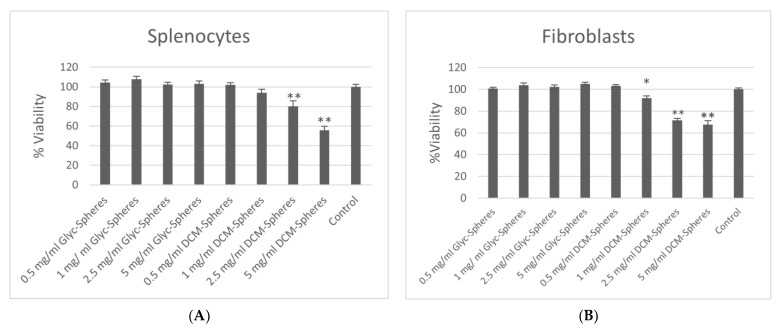
Alamar Blue assay to determine cytotoxicity of PLGA spheres made from DCM and GPI method on splenocytes (**A**) and fibroblast (**B**). * indicates *p* < 0.05, ** indicates *p* < 0.01 compared to control (cells with no PLGA spheres).

The potential toxicity of microspheres prepared with either DCM or GPI methodology was assessed and compared by measuring the effect of each type of sphere on the viability of fibroblasts or Con A stimulation of mononuclear spleen cells using an Alamar Blue Assay (Figure 6). The proliferation of spleen cells and fibroblasts significantly and dose-dependently decreased with the number of incubated DCM-constructed spheres. All doses of glycofurol-constructed (GPI) PLGA spheres caused no decrease in viability of fibroblasts and spleen cells.

### 3.5. DEX Release from PLGA Microspheres

**Figure 7:**
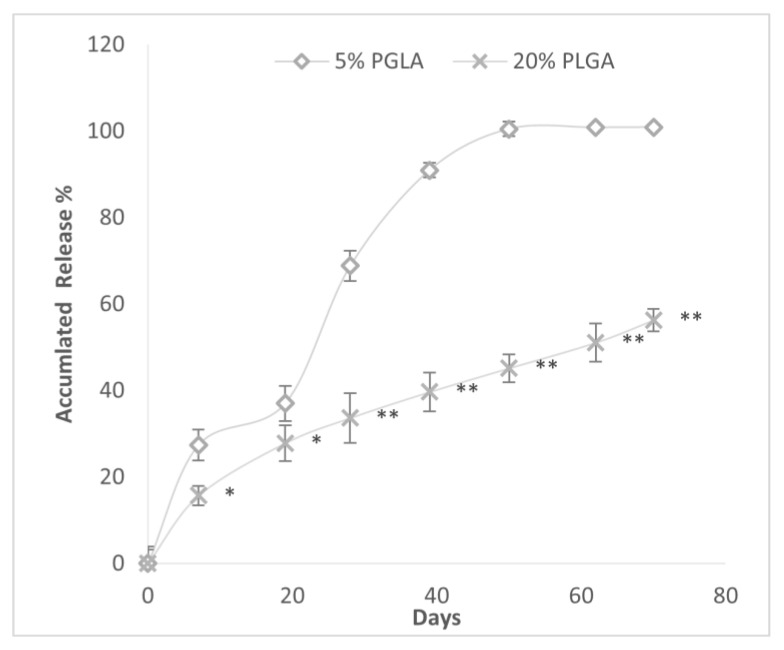
Accumulated drug release (%) over time from PLGA microspheres constructed with either 20% or 5% PLGA and 20% DEX by GPI method. * *p* < 0.05; ** *p* < 0.0001 vs. 5% PLGA.

The effect of PLGA concentration within GPI-prepared spheres on the release of DEX is depicted in Figure 7. Spheres constructed with 5% PLGA had a faster and greater mean cumulative DEX release at every time point. The time (days) to 50% accumulated drug release was lower in the 5% vs. 20% PLGA microspheres, 23.67 (4.7) vs. 61.8 (7.4) days *p* < 0.001. The duration of drug release was shorter in the 5% PLGA than 20% PLGA spheres.

**Figure 8:**
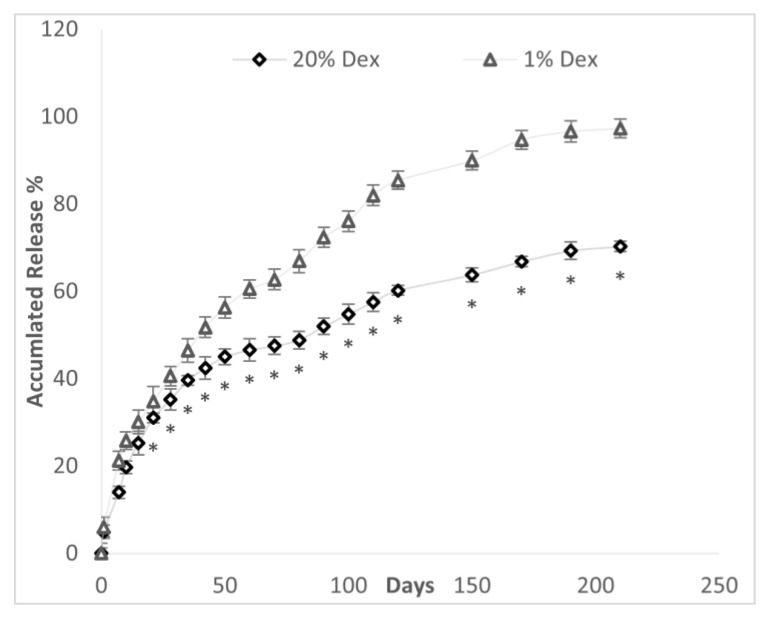
Accumulated drug release (%) over time from PLGA microspheres made from a 20% PLGA solution constructed with a drug load of 20% and 1% DEX with GPI method. * *p* < 0.02 compared to 1% DEX microspheres.

The effect of DEX concentration on drug release from 20% PLGA microspheres is depicted in Figure 8. The drug bursts in the 20% DEX and 1% DEX microspheres were similar, 5.99% and 4.91%, respectively (Table 3). In both the 1% and 20% DEX-made spheres, there was no a lag phase after the drug burst. The time (days) to 50% accumulated drug release was lower in the 1% vs. 20% DEX microspheres, 44.7 (5.2) days vs. 71.5 (5.3) days (*p* < 0.001). Both the 1% and 20% DEX spheres had a sustained release rate over 6 months.

The pattern of Dex release was assessed in GPI-constructed spheres made with 1% and 20% Dex. The pattern of drug release included a small decline in drug release rate from 48–68 days in the 20% DEX spheres and a very much smaller decline of drug release from 60–75 days in the 1% DEX spheres. After the burst until halfway through the study at 110 days, the 1% DEX sphere exhibited a tighter zero-order fit than the 20% DEX spheres with a mean R^2^ of 0.971 (0.015) (range of 0.96–0.98) vs. 0.884 (0.033) (0.85–0.93), respectively (*p* < 0.0002). From 110 days to the end of the study at 210 days, the DEX 1% and DEX 20% microspheres exhibited similar zero-order kinetics with an R^2^ of 0.920 (0.023) (0.90–0.95) and 0.941 (0.052) (0.94–0.99). Using different mathematical models, the assessment of drug release from 1% and 20% DEX spheres for the entire length of the experiment (0–210 days) is described in Table 4.

**Figure 9:**
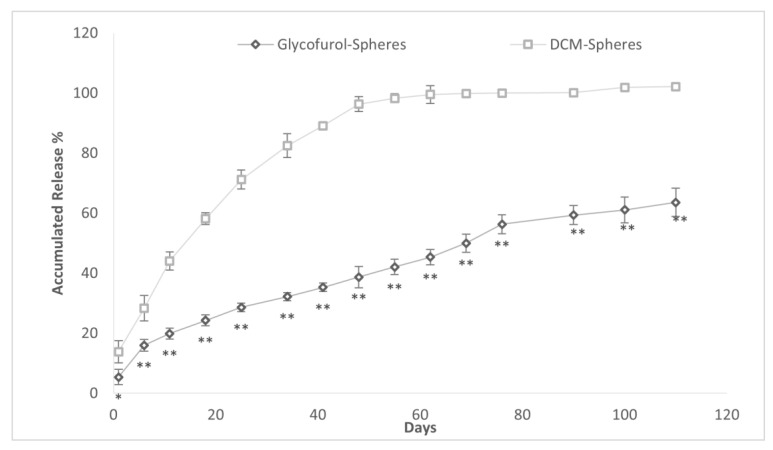
Accumulated DEX release (% ±SD) from 20% DEX and 20% PLGA spheres made from GPI method and DCM method. * indicates *p* < 0.05 vs. DCM, ** indicates *p* < 0.001 vs. DCM.

The accumulated DEX release over time was compared in PLGA microspheres made with either GPI method or DCM methodology (Figure 9). The mean (SD) drug burst was greater in the DCM spheres than the glycofurol (GPI) spheres (13.86 (4.12)% vs. 5.43 (1.67)%; *p* < 0.05). The time (days) to 50% accumulated drug release was lower in the DCM vs. glycofurol PLGA microsphere, 12.1 (1.9) days vs. 71.1 (6.0) days *p* < 0.0001. The DCM-prepared spheres released all of the releasable drug within 55 days as compared to >110 days in the glycofurol spheres when the experiment was terminated.

We assessed if the prolonged DEX drug release from GPI-constructed spheres was specific for that drug only. PLGA spheres were constructed with the GPI methodology loaded with a very different substance than DEX (log P 1.83), Sudan l11, a lysochrome fat soluble dye with a Log P of 5.8. We also compared these Sudan-111-loaded GPI-constructed spheres with PLGA spheres prepared with glycofurol but using a different method, a glycofurol extraction method as described by Allhenn [32].

The encapsulation efficiency of 20% Sudan-111-loaded PLGA spheres prepared using GPI method (73.11% (2.34)) was significantly higher (*p* < 0.001) than the glycofurol extraction method (GEM) (34.91% (3.98)) (Table 3).

**Figure 10:**
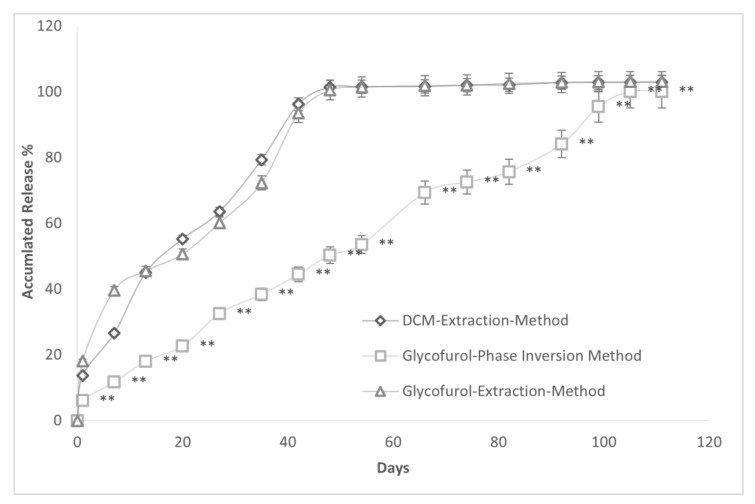
Accumulated drug release (mean (±SD)%) over time from PLGA microspheres constructed with 20% PLGA and 20% Sudan 111 prepared using either DCM emulsion/evaporation method, glycofurol extraction method (GEM), or GPI methodology. The mean diameter (SD) of spheres prepared by DCM, GEM, and GPI methods were 369.14 (25.32) μm, 313.24 (41.25) μm, and 357.45 (34.85) μm, respectively. ** *p* < 0.0001 compared to DCM extraction method and GEM.

The release of Sudan 111 from spheres constructed with solvent extraction with DCM and the GEM of Allhenn methodologies were very similar (Figure 10). Further, Sudan 111 release from spheres constructed with our GPI methodology was far slower than Sudan 11 release from spheres made by both DCM and GEM methods. The time (days) to 50% accumulated drug release was lower in the DCM and GEM spheres vs. the GPI microsphere, 16.91 (2.24) days and 19.84 (1.58) vs. 46.81 (2.12) days *p* < 0.0001.

The duration of release was approximately 45 days using the extraction methods while the release was more than twice as long in spheres using our GPI methodology.

## 4. Discussion

We developed a modified phase inversion method using the non-toxic solvent glycofurol to construct DEX-laden PLGA spheres that release drugs over an extended period of time.

Utilizing an air-driven droplet generator device to deliver the inner phase into the outer aqueous phase, we found the optimal device settings to construct PLGA microspheres with 20% PLGA were a solvent/drug pump flow rate of 5 µL min^−1^, a 35-gauge needle, an air flow rate of 30 LPM, a d1 at 2.5 mm, and a d2 at 25 mm.

Very small microspheres were not attainable with this exact method. However, modifications of our procedure such as spraying solvent drug mixture using acoustic excitation or using a non-solvent carrier stream may allow this [43].

Previous reports show that PLGA microspheres made with a DCM emulsion–evaporation technique or with electrostatic methodologies commonly have a wide distribution of sphere sizes. One standard deviation of this size distribution frequently equaled 50% [30] making the procedure less amenable for clinical use. However, spheres prepared with our GPI methodology possessed a much tighter distribution of spheres sizes with one standard deviation of the distribution equaling only 10% of the mean sphere’s diameter.

The encapsulation efficiency of DEX within the spheres was very high (80%), resulting in a much greater DEX load of 16% than the previously described loads of 1–8% [19,44,45,46]. These higher drug loads can potentially provide a much larger depot of drug per PLGA mass within the body, which would allow the potential release of the drug over a longer period of time.

One of the problems utilizing spheres prepared with organic solvents, such as dimethylchloride, has been the toxicity to cells in the surrounding tissue and the degrading effect on the drug payload [47]. Similarly, we found that PLGA microspheres made with DCM were toxic to spleen cells and fibroblasts in vitro. However, PLGA spheres made with glycofurol were non-toxic, having no inhibitory effect on either target cell, a significant advantage for using this type of sphere.

Hickey observed that the DEX molecule released from PLGA spheres constructed with DCM degraded within just a week of in vitro incubation [19]. Their HPLC studies revealed a second peak, representing degraded DEX, which increased in amplitude over time [19]. In contrast, the glycofurol-constructed spheres by GPI caused no DEX degradation even after 5 weeks of incubation.

Although glycofurol is considered to be of low toxicity as it is classified as a Class 1 solvent by the FDA, it seems reasonable to keep the amount of solvent within the spheres as low as possible. The residual amount of glycofurol within the spheres presented here was 5.71 mg/100 mg, much less than the 14–16.9 mg/100 mg found in the PLGA spheres previously described [32,33]. This residual amount may be further reduced by utilizing standard methods such as by dialysis [31].

There are few previous reports describing the release of drugs from PLGA spheres constructed with glycofurol without the addition of the volatile organic solvents. All these previous methodologies differed with ours. Further, these previously described PLGA spheres were unable to release drugs over an extended period of time.

Allhenn constructed microspheres with glycofurol using an emulsion extraction method (GEM) and incorporated three individual substances, ritonavir, lopinavir and Sudan lll. The maximal duration of release was only 4 h, 20 h, and 18 days, respectively [32]. Kim composed PLGA nanospheres using interfacial polymer deposition method with glycofurol and then loaded paclitaxel by adsorption on to the sphere. However, all of the drug was released by 7 days [31]. Using a phase separation method, Swed constructed nanospheres with glycofurol incorporating lysozyme or TGF-b with a total duration of drug release of only 10 days and 20 days, respectively [30]. Conversely, the duration of DEX release from microspheres prepared with glycofurol in the present report exceeded 6 months, thus making these spheres more amenable for long-term therapy.

There are no previous reports describing DEX-laden microspheres constructed with glycofurol. Therefore, we assessed if the results of extended duration of Dex release was due to the different drugs that were encapsulated. Our studies demonstrated that the specific compound that was encapsulated, DEX, was not the reason for the prolonged drug release in our glycofurol-generated spheres. This is because we found that the duration of Sudan 111 release from PLGA spheres constructed using our phase inversion methodology was far longer than the release of Sudan 111 from spheres we constructed with the GEM method and those constructed by GEM reported by Allhenn [32]. Further, the long duration of Sudan 111 release from spheres prepared with the GPI methodology reported herein was similar to the long duration of DEX release in our similarly constructed spheres. Future studies will be performed to assess the differences between DCM- and glycofurol-constructed spheres (GPI) using a wide range of Dex concentrations.

The kinetics of drug release from PLGA spheres is described to generally occur in three phases [48]. An initial short drug burst over the first 24 h. is thought to be due to drug release from the surface or just below the surface of the microsphere. Then, a minimal drug release for 2–3 or more weeks usually occurs, called a lag phase, which is thought to be due to the drug diffusing from the core of the sphere to the surface. Spheres with very short duration of drug release (less than 1–2 weeks) may not demonstrate this lag phase. The lag phase is frequently followed by a third phase of a more rapid rate of drug release sometimes occurring at a constant (zero-order kinetics) induced by the hydrolytic breakdown of the PLGA.

The kinetics of drug release from our DEX-laden spheres prepared by GPI are very different from previous reports using DCM. First, the glycofurol-constructed spheres have relatively small bursts of 5% to 6% as compared to the 18% to 65% bursts that have been previously reported in DEX spheres constructed with DCM [18,19,46]. Similarly, the burst in DCM-prepared spheres in our study was greater than that of the glycofurol-prepared spheres. A small burst may be clinically helpful to avoid a large bolus of drug. The low burst that we observed could be due to smaller amounts of drug on the sphere surface and/or a very slow time for the drug near the surface of the spheres to diffuse through the pores to the surface. We do not expect the later explanation since tiny pores below the surface would most likely result in a lag phase after the burst that did not occur in these spheres. Gel coating of our spheres may be able to further decrease the burst phase [18].

After the burst, the drug release patterns of the 20% DEX- and 1% DEX-constructed spheres by GPI were very different. The rate of accumulated drug release (%) was lower from the 20% DEX spheres than from the 1% DEX spheres. This was not unexpected since slower rates of drug release have been previously observed in PLGA spheres with higher loads of hydrophobic drug. This is thought to be due the crystallized or aggregated drug that frequently occurs in higher hydrophobic drug loads than in the more dispersed drug spheres with lower hydrophobic drug loads [49].

The small declines in rates of drug release that occurred from 60–75 days and 48–68 days in the 1% DEX and 20% DEX spheres, respectively, could be due to the lack of releasable drug near the sphere surface. It could also be due to the time needed to transport drug from the core of the sphere to the surface, which is called the lag phase. Alternatively, the increased DEX release after the slowdown may solely be due to hydrolytic degradation of PLGA. This then starts the last phase of drug release, a phase of brisk extended drug release at zero-order kinetics in both the 1% and 20% DEX spheres.

The kinetics of DEX release for the first half of the experiment, ending at 110 days, also differed between the DEX 1% and 20% spheres with only the 1% DEX spheres exhibiting drug release at zero order. However, from 110 days to the end of the experiment, drug release followed the zero-order kinetics from both the 1% DEX and 20% DEX spheres. This zero-order release is frequently found in the last phase of drug release from PLGA spheres, the phase of hydrolytic breakdown of the spheres. The zero-order kinetics of DEX release from the GPI-constructed sphere of over 110 days duration is the longest ever reported from PLGA spheres. This prolonged zero-order kinetic drug release is frequently clinically advantageous for consistent drug release.

The degree erosion vs. diffusion mechanism of drug release was assessed by calculating the diffusion exponent A and erosion exponent B derived from the non-linear fitted Kopchas model. The calculated value of A and B indicate that both factors, diffusion and erosion, are responsible for drug release [42]. The higher value of A/B 5.6 and 8.2 for the 1%- and 20%-DEX-constructed glycofurol spheres, respectively, indicate that the predominant mechanism of drug release is from bulk diffusion vs. erosion.

When assessing the kinetics of drug release over the entire time of the experiment (0–210 days) using R^2^, MSC, and AIC, the best fit mathematical model was the Weibull model [50] in both the 1%-DEX- and 20%-DEX-constructed spheres. The Korsmeyer–Peppas model [51] equally fit with the Weibull model to describe drug release from the 1% DEX spheres.

The major mechanism of drug release over the entire period of release appears to be Fickian diffusion. First, the n exponents of the Korsmeyer–Peppas equation for the 1% DEX (b = 0.43) and 20% DEX (b = 0.40) spheres supported a mechanism of Fickian diffusion (b < 0.45). Second, the b exponents of the Weibull equation for the 1% DEX and 20% DEX spheres were 0.75 and 0.57, supporting Fickian diffusion of DEX release in the 1% DEX and Fickian, plus another release pathway for the 20% DEX spheres. In the equation Fickian is present when b < 0.75 and a diffusion plus another release pathway is present when b > 0.75.

Conversely, the mechanism of DEX release from the 20% DEX spheres constructed with DCM was more complex than the GPI spheres with a higher n exponent (n = 0.616) of the Korsmeyer–Peppas equation and higher b exponent (b = 0.934) of the Weibull equation (*p* < 0.05 and *p* < 0.01, respectively).

The k constants from the Korsmeyer–Peppas equation in the 1%DEX and 20% Dex GPI spheres of 9.9 and 8.6, respectively, were not significantly different, suggesting that the structure and geometry of carrier systems were similar.

An extended duration of drug release from implanted microspheres may be critical in treating chronic inflammation and diseases such as cancer, chronic inflammatory bowel disease, arthritis, chronic abscess, and AIDS. The duration of DEX release from our 1% and 20% DEX microspheres was approximately 6 months, far longer than the duration of active release of previously reported DEX-laden PLGA spheres prepared with DCM [18,19,46]. Further, when we compared DEX release from large-sized DCM- and GPI-prepared PLGA spheres constructed with similar PLGA types, PLGA molecular weights, PLA and PLG proportions, and concentrations of PLGA and DEX, the GPI-prepared spheres had a far longer duration of drug release than spheres constructed with DCM. For example, the amount of time to achieve 50% drug release was 12 days vs. 71 days in DCM and GPI spheres, respectively. This is consistent with the duration of DEX release from DCM-prepared PLGA spheres that were previously reported [52]. Of course, alterations to our methodology to prepare spheres, such as encasing our microspheres in gels may also improve and extend the release of drug as previously been shown [53].

The extended duration of DEX release from the GPI-constructed PLGA microspheres as compared to DCM spheres may be related to the more rapid speed that the more polar solvent, glycofurol as compared to DCM, diffuses out of the polymer (PLGA) and into the outer aqueous phase during sphere preparation. This, in turn, results in a rapid formation of the spheres that has been previously shown to decrease water diffusion into PLGA spheres [54]. Such a decrease in water diffusion can, in turn, slow PLGA degradation and lengthen the duration of drug release. This explanation is supported by our observation that the spheres form very rapidly and water diffusion into the GPI PLGA spheres was much slower than the water diffusion into the DCM-made PLGA spheres. This slower water diffusion into these spheres constructed with GPI methodology appeared not to be due to an increase in porosity, density, or innate sphere hardness of the glycofurol spheres, since these physical characteristics were in fact greater in the DCM-constructed spheres.

Similarly, the extended release of DEX that was observed when higher concentrations of PLGA were utilized in sphere construction may be a result of the more rapid formation of the spheres and resultant decreased water diffusion.

With such the rapid microsphere formation, solvent residues could be present on the surface of the spheres. To detect the presence of a non-specific residue on the sphere surface, scanning electron microscopy may be useful. Different forms of spectroscopy, such as photoelectron spectroscopy, may further be helpful in assessing the specific surface composition/residue.

By understanding the effects that PLGA concentration and drug concentration have on the kinetics of DEX release from the glycofurol-constructed PLGA spheres as reported herein, one may be able tune the rate of drug release. This tuning procedure may be further improved in the future by determining the effects of different molecular weights of PLGA and proportions of PLA to PGA on the release rate of drugs [16].

## 5. Conclusions

We have constructed PLGA microspheres using the non-toxic solvent glycofurol with a modified phase inversion method (GPI) that can release DEX or Sudan 111 over a prolonged period of time. These spheres are approximately 385 μm in diameter and have a low glycofurol content as compared to previously made spheres. With the optimal PLGA concentration of 20%, the drug encapsulation efficiency is high at 80%, allowing large drug loads, which is clinically advantageous. Unlike spheres made with the DCM emulsion methodology, we found spheres generated with GPI are not toxic to target cells in vitro and do not denature the DEX molecule as found with DCM-prepared spheres. The duration of DEX release is approximately 6 months, far longer than previously described spheres. This extended release may be related to the decreased diffusion of water into these spheres. The prolonged DEX release from GPI-constructed spheres was not specific to the drug DEX, since both Sudan lll and Dex-laden PLGA spheres constructed with the GPI process had similar and prolonged drug release. The pattern of drug release can be tuned with altering the concentration of Dex and PLGA. Further, the kinetics of release are also more clinically advantageous with a smaller burst and a prolonged time of constant drug release rate, particularly in the spheres made with 1% DEX.

## Figures and Tables

**Table 1:** The morphology and size (diameter) of PLGA microspheres resulting from methodologic alterations of PLGA concentration, needle gauge, distance of needle exposed past below the air tube, distance of needle to aqueous phase, and air flow rate. * indicates the parameters selected for preparing PLGA spheres for the drug elution experiments.

% PLGA	Needle Exposed, d1 (mm)	Needle Gauge	Distance Needle to Surface Water, d2 (cm)	Air Flow (LPM)	Morphology/Size of Spheres
20	2	27	2.5	30	Sphere, 300–400 µm *
20	5	27	2.5	30	Sphere, 400–600 µm
20	2	27	2.5	15	Sphere, 500–700 µm
20	2	27	2.5	45	Rods
20	2	27	5	30	Sphere, 300–400 µm
20	2	30	2.5	30	Rods & Sphere, 300–400 µm
5	2	27	2.5	30	Sphere, 300–400 µm *
5	5	27	2.5	30	Sphere, 350–600 µm
5	2	27	2.5	15	Sphere, 400–650 µm
5	2	27	2.5	45	Rods
5	2	27	5	30	Sphere, 300–400 µm
5	2	30	2.5	30	Rods & Sphere, 300–400 µm
1	2	27	2.5	30	Sphere, 300–400 µm *
1	5	27	2.5	30	Sphere, 300–500 µm
1	2	27	2.5	15	Sphere, 400–600 µm
1	2	27	2.5	45	Rods
1	2	27	5	30	Rods and Sphere, 300–400 µm
1	2	30	2.5	30	Rods and Sphere, 300–400 µm

**Table 2:** The effect of PLGA concentration on the solidification time and hardness (compressive modulus) of PLGA microspheres constructed with glycofurol. The mean time required for spheres to turn opaque following immersion of the ‘organic” phase droplets into the aqueous phase was compared in 20% PLGA, 10% PLGA, 5% PLGA, and 1% PLGA spheres (n = 12). Mean sphere hardness was also assessed (n = 30/group). Mean solidification time and mean compressive modulus were significantly different between the PLGA groups (*p* < 0.001 and *p* < 0.003, respectively).

% PLGA	Sphere Solidification Time (t_1_) (s)	Compressive Modulus E (×10^−3^ mPa)
20%PLGA	3.83 (0.15)	18.44 ± 0.733
10% PLGA	7.33 (0.38)	9.212 ± 0.784
5% PLGA	10.00 (0.33)	6.468 ± 0.48
1% PLGA	12.83 (0.28)	4.116 ± 0.392

**Table 3:** Density, porosity, encapsulation efficiency%, and drug burst from PLGA spheres constructed by glycofurol phase inversion method (GPI), emulsion/evaporation technique with DCM, and glycofurol extraction method (GEM).

	Density (g/cm^3^)	Porosity (Φ)	Encapsulation Efficiency	Burst Release% (24 h)
No drug–20% PLGA–GPI	0.170 (0.005)	0.8692 (0.004)	-	-
20% DEX 20% PLGA–GPI	0.196 (0.009)	0.8493 (0.008)	80.45% (1.23)	4.88% (0.16)
1% DEX 20% PLGA–GPI	0.163 (0.008)	0.8749 (0.007)	86.21% (2.87)	5.99% (0.17)
20% DEX 5% PLGA–GPI	0.133 (0.011)	0.9073 (0.010)	47.25% (3.74) d	15.68% (2.12) b
No drug 20% PLGA–DCM	0.217 (0.006) e	0.8333 (0.007)	-	-
20% DEX 20% PLGA–DCM	0.205 (0.009)	0.8423 (0.012)	72.25% (1.45) d	28.65% (3.34) b
20% Sudan III–20% PLGA–DCM	0.228 (0.012)	0.7147 (0.009) a	62.41% (2.84) f	19.28% (4.41) c
No drug–20% PLGA–GEM	0.345 (0.004) e	0.7348 (0.003)	-	-
20% Sudan III–20% PLGA–GEM	0.331 (0.002)	0.7448 (0.002) a	34.91% (3.98) f	18.17% (3.79) c
20% Sudan III–20% PLGA–GPI	0.178 (0.007) g	0.8630 (0.005)	73.11% (2.34)	6.03% (1.02)

‘a’ *p* < 0.01 compared to 20% Sudan III 20%PLGA–phase inversion, ‘b’ *p* < 0.005 compared to 20% DEX 20%PLGA–phase inversion, ‘c’ *p* < 0.001 compared to 20% Sudan III 20%PLGA–phase inversion, ‘d’ *p* <0.01 compared to 20% DEX 20%PLGA–phase inversion, ‘e’ *p* < 0.01 compared to 20% DEX 20%PLGA–phase inversion, ‘f’ *p* < 0.01 compared to 20% Sudan III 20%PLGA–phase inversion, ‘g’ *p* < 0.01 compared to Sudan 111 PLGA–GEM and Sudan 111–DCM.

**Table 4:** Comparison of kinetic models of drug release for 20% DEX vs. 1% DEX. The ‘n’ exponent of the Korsmeyer–Peppas equation for the 1% and 20% DEX were similar at 0.43 and 0.40 and the ‘b’ exponent of the Weibull equation for the 1% and 20% DEX were 0.75 and 0.57. The ‘k’ drug release constant of the Korsmeyer–Peppas equation were similar in the 1% DEX and 20% Dex spheres, 9.9 and 8.6, respectively.

Model	20%DEX-Day 0 to 210			1% DEX-Day 0 to 210			
	Avg. R^2^	Avg. AIC	Avg. MSC	Avg. R^2^	Avg. AIC	Avg. MSC	
**First Order**	0.7721 (0.0689)	148.35 (4.9)	1.43 (0.34)	0.9318 (0.0395)	133.78 (7.46)	2.75 (0.57)	
**Gompertz**	0.9828 (0.0023)	98.35 (3.83)	3.93 (0.14)	0.9297 (0.0159)	138.11 (8.54)	2.53 (0.22)	
**Higuchi**	0.9492 (0.0193)	117.57 (7.32)	2.97 (0.44)	0.9685 (0.0175)	117.64 (11.09)	3.55 (0.69)	
**Hixson-Crowell**	0.6584 (0.0862)	156.79 (3.68)	1.01 (0.27)	0.8794 (0.0684)	144.92 (8.23)	2.19 (0.61)	
**Korsmeyer-Peppas**	0.9825 (0.0031)	98.57 (3.95)	3.92 (0.19)	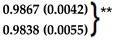	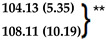	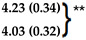	
**Weibull**	0.9921 (0.0029) *	81.44 (7.33) *	4.77 (0.41) *				
**Zero-Order**	0.2574 (0.1254)	172.74 (2)	0.21 (0.17)	0.3754 (0.1313)	180.91 (2.86)	0.39 (0.22)	
	**A**	**B**	**A/B**	**A**	**B**	**A/B**	**Mechanism**
**Kopcha’s**	0.201 (0.007)	0.024 (0.002)	8.26	0.16 (0.008)	0.028 (0.006)	5.61	Diffusion (A/B > 1)

Best fit model(s) for 20% Dex was Weibull and Korsmeyer-Peppas and for 1% Dex was Weibull. * *p* < 0.005 ** *p* < 0.008 vs. all other models.

## Data Availability

Data are contained within the article.
